# 100 m soil landscape grids of Canada

**DOI:** 10.1038/s41597-025-05460-4

**Published:** 2025-07-10

**Authors:** Xiaoyuan Geng, Juanxia He, Victoria Grima, Yefang Jiang, Maggi Tetreau, Stephen Crittenden, Simon Kiley, Albert. J. VandenBygaart, Jason Vanrobaeys

**Affiliations:** 1 Science and Technology Branch, Agriculture and Agri-Food Canada, Ottawa, Canada; 2 Swan Lake First Nation, Swan Lake Land Governance Office, Swan Lake, R0G 0B9 Manitoba Canada; 3 Parks Canada, P.O. Box 150, Val Marie, S0N 2T0 Saskatchewan Canada; 4 Pest Management Regulatory Agency, Health Canada, Ottawa, Canada

**Keywords:** Carbon cycle, Element cycles

## Abstract

With the latest collection of soil point and available co-variables data, 100 m grids of soil type and selected soil attributes of Canada are developed. While model-based statistical validations during the machine learning runs are satisfactory, limited independent statistical validations are also made. The 100 m soil landscape grids can be used for national and regional soil organic carbon stock inventory and carbon sequestration assessment. There are also potential applications for soil health, soil erosion, land suitability and other large area-based modelling and applications.

## Background & Summary

Canada is the second largest country in the world with nearly 10 million square kilometres of land area, approximately 62 million hectares of which is agricultural land. Up-to-date soil landscape data are essential for precision agriculture, ecosystem and nutrient cycling modelling, soil organic carbon (SOC) stock inventory, SOC sequestration potential assessment, and sustainable soil management use^1^. With diminishing investment in conventional soil surveys, operational predictive soil mapping (PSM) frameworks and methods have been used to provide up-to-date soil data and information across landscape scales in Canada^2,3^. Predictive soil mapping, also referred to as digital soil mapping (DSM), is the computer-assisted production of spatial data or maps of soil types and soil properties using structured knowledge of soil and its relation with environmental variables. PSM involves the creation and population of spatially explicit soil information from field and laboratory observations coupled with spatial and non-spatial soil inference systems^4^. Soil classes and properties include categorical examples such as soil classification names, and interdependent continuous variables such as bulk density. In Canada, the first gridded national soil and soil landscape data developed using the PSM method were the subset of the global PSM outputs^5^. One of the advantages of PSM methods is that predictions can be repeated or updated as new training point data and co-variable data become available. Because of that, the global soil grids were updated in 2021^6^. When computing capacity is available, ensemble machine learning can improve the performance and can provide better deterministic predictions of soil types and properties^7^. Among the studied machine learners such as artificial neural network, support vector machine, gradient boosting decision tree and random forest algorithms, different variations of random forest algorithms have produced more accurate PSM outcomes^3,5^.

In Canada, with more available soil point data, new co-variable data and advances in machine learning algorithms, regional data of PSM have also been generated^8,9^. For the nationwide PSM, more point soil data are being gathered and compiled^10^. Bias correction^11^ has been added to the random forest-based machine learning algorithms. Along with new co-variable data, nationwide PSM has been conducted at a 100 m grid resolution. These kinds of incremental predictive soil mapping operations will be repeated in the future as more nationwide soil sampling work yields new data based on purposive sampling designs^12^.

The outputs of PSM should be accompanied with uncertainty measures^13^ and independent accuracy assessment^12,14^. PSM output accuracy assessment remains a challenge, especially when validating the PSM outputs at regional, national and global scales^15^. Point-to-point based validation requires a purposefully-designed and collected independent point data set. For large mapped areas, this requirement is often met with using legacy soil survey point data that may not adequately provide suitable spatial representability due to the variability of landscapes and processes that influence soil properties. Mapped data for large areas like USA and Australia, point data-based independent validations often come with lower accuracies^16,17^. However, areal or management unit-based accuracy assessments are more meaningful to end users of the PSM outputs^18^. The primary goal of this study is to develop 100 m soil landscape grids of Canada using PSM method, and to conduct statistical evaluations of the predictions^19^.

## Methods

### Machine learning and predictive soil mapping

Soil classes and properties are influenced by soil forming factors, namely climate, topography, parent material, organisms and time. This is further described with a state factor equation: CLORPT (CL = climate condition at a point; O = organisms including land cover; R = relief or topographic attributes; P = parent or surficial geological material; T = time or age) and is expressed as a theoretical soil-landscape relationship^20^. To truly reflect the relationship between soil properties and soil forming factors, the universal model of spatial variation recognizes that both the deterministic and stochastic components of soil forming factors need to be modelled^21^. To represent the soil forming factors using computerized systems, McBratney *et al*.^22^ expanded the CLORPT to SCORPAN model^22^. SCORPAN model states that soil class or soil property is function of soil intrinsic properties, climate, organism, relief, parent materials, time and spatial location.

1 $${\boldsymbol{S}}={\boldsymbol{f}}\left({\boldsymbol{s}},{\boldsymbol{c}},{\boldsymbol{o}},{\boldsymbol{r}},{\boldsymbol{p}},{\boldsymbol{a}},{\boldsymbol{n}}\right)$$

Where

s - soil intrinsic properties

c - climate

o -organism

r - relief or topography

p - parent materials

a - age or time

n - spatial coordinates of a point soil data

**Fig. 1:**
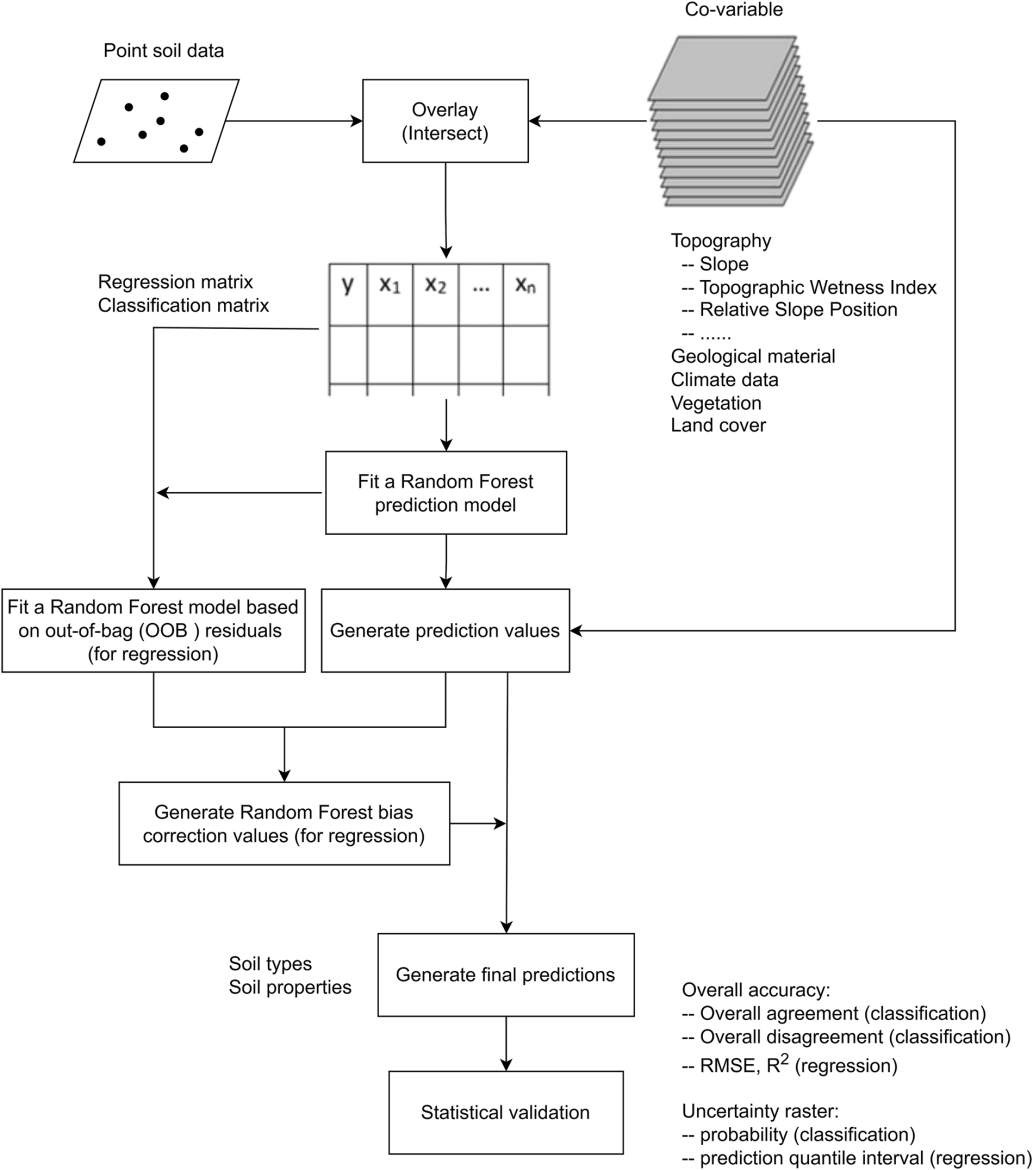
Flow chart of predictive soil mapping. Key steps of machine learning based predictive soil mapping.

Such soil-environment relationship frameworks, especially the SCORPAN model, have been the foundation of recent predictive soil mapping^5^. The key steps of predictive soil mapping include training data, co-variable data collection and compilation, machine learning model building, predictions, uncertainty measures, intrinsic and independent statistical validations, and data quality assurance (Fig. 1). R package Ranger was used to construct random forest-based inference models and predictions of great group soil classes and selected soil properties^23,24^. For soil property inference, the use of quantile random forest (QRF) option enabled the estimation of prediction uncertainty.

#### Point soil data processing and compilation

**Fig. 2:**
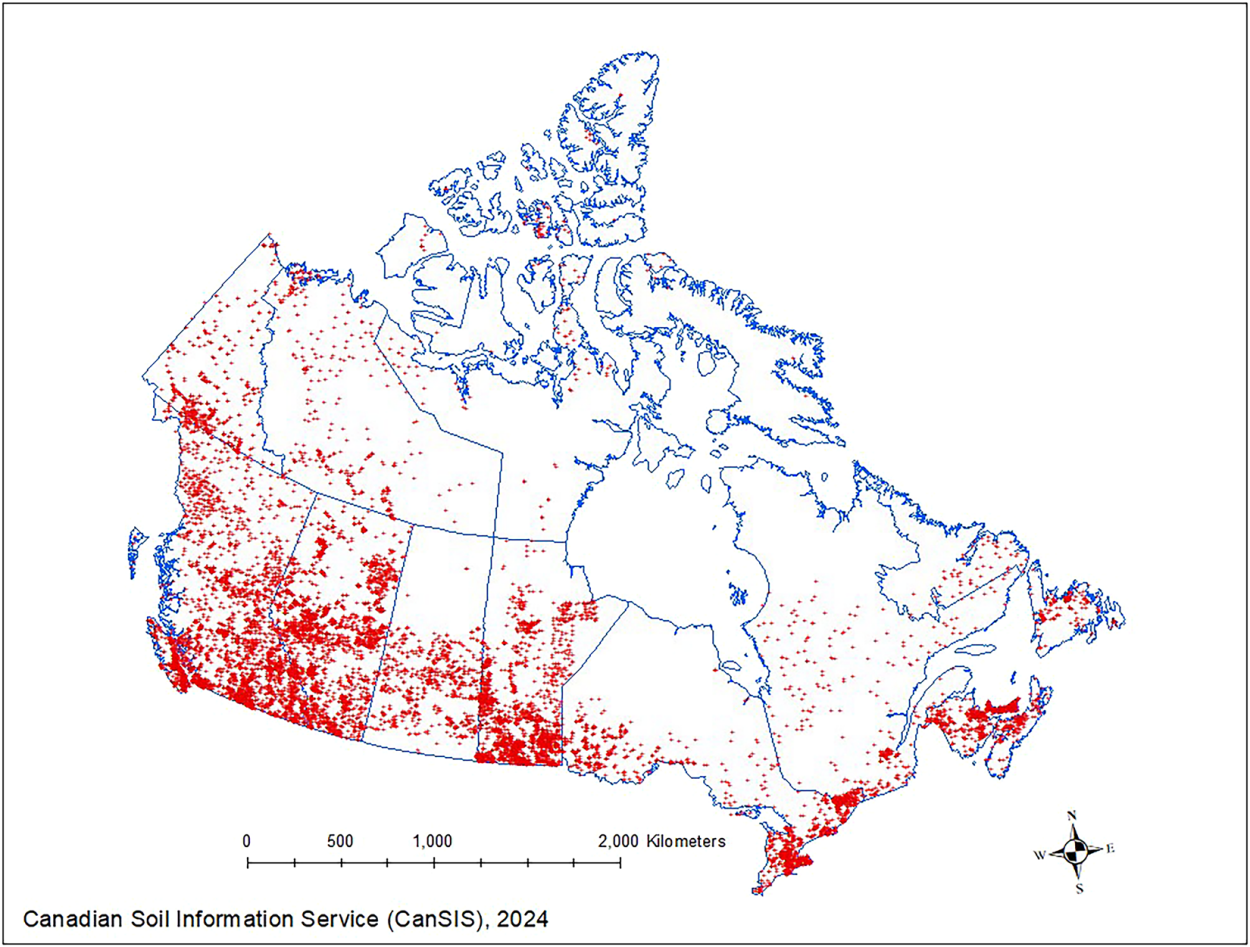
Point soil data and distribution. Available soil point or pedon data were dominantly collected between 1980 to 2000. The data are clustered within agriculture region of Canada and not evenly distributed across the nation.

Point soil data with geographic coordinates are the key training data source for machine learning. A point of soil from a specific location is referred to as a pedon. A standard pedon “is the smallest, three-dimensional unit at the surface of the earth that is considered as a soil”^25^, and it usually has a surface area of approximately 1 m^2^. There is a need to harmonize the layered soil property values to provide uniform depths^26^ because the original point soil data were not collected from consistent depths. Spline algorithms implemented in R^23^ were used to harmonize point soil data to uniform depths^27^. For this work, the two main soil point data sets were from Canadian Soil Information Service (https://sis.agr.gc.ca/cansis/nsdb/npdb/index.html) and Canadian Forest Service^10^. Figure 2 shows the spatial location and distribution of the combined point soil data used for this project. Most of the sampled pedons were located in the southern regions of Canada where land used for agriculture was located. The locations of the legacy pedons were often selected based on ease-of-access and access permission, therefore the distribution of those pedons was skewed and clustered. The great group soil class names used in the point soil data set were harmonized based on the third edition of the Canadian Soil Classification System^25^.

#### Co-variable data processing and compilation

The co-variables of PSM work include climate, land cover, topography and surficial geology data. Within the topographic theme, 100 m grid size topographic co-variables such as Iwahashi and Pike landform classes^28^ and Multi-resolution valley bottom flatness^29^ were derived using the 16 m grid size digital elevation model (DEM) data and SAGA GIS^30^. Table 1 further summarizes all 70 co-variable layers used for this work.

**Table 1:** Co-variables used for 100 m soil data prediction.

Co-variable description	Unit
Monthly mean (Jan-Dec) maximum temperature from^38^ 1958 to 2020	°C
Monthly mean minimum temperature (Jan-Dec)^38^ from 1958 to 2020	°C
Monthly mean accumulated precipitation (Jan-Dec)^38^ from 1958 to 2020	mm
Annual mean precipitation based on monthly accumulated precipitation^38^	mm
Land surface temperature day-time monthly mean^39^ (Mar.–Nov, 2000–2017)	°C
Land surface temperature night-time monthly mean^39^ (Mar.-Nov, 2000–2017)	°C
Monthly mean of MODIS (MOD13Q1) NDVI (Mar. – Sept. 2000–2021)^40^	
MOD13Q1 NDVI^40^ for 15–30 August, 2020	
MOD13Q1 NDVI^40^ for 1–15 July, 2020	
Monthly mean Band 7 of MCD43A4 (Mar.- Sept. 2000–2021)^40^	
Highest NDVI of 2020 from Landsat 8 (https://developers.google.com/earth-engine/datasets/catalog/LANDSAT_LC08_C02_T2_L2), U.S. Geological Survey	
Land cover of 2020 (https://www.cec.org/north-american-land-change-monitoring-system)	
Elevation (https://open.canada.ca/data/en/dataset/7f245e4d-76c2-4caa-951a-45d1d2051333. Licence: CC?BY?4.0.)	m
Elevation derived landform classes^28,30^	
Elevation derived multiresolution index of the ridge top flatness (MRRTF)^29,30^	
Elevation derived multiresolution index of valley bottom flatness (MRVBF)^29,30^	
Elevation derived plan curvature^30^	
Elevation derived profile curvature^30^	
Elevation derived slope gradient^30^	radians
Elevation derived topographic position index^30^	
Elevation derived valley depth^30^	m
Elevation derived relative hill slope position^30^	
Elevation derived topographic wetness index^30^	
Depth to bedrock (https://open.canada.ca/data/en/dataset/83f37aa2-e362-f942-0ad0-de45c850b5b9)	m
Surficial geological materials^41^	

#### Data tiling and parallel computing

**Fig. 3:**
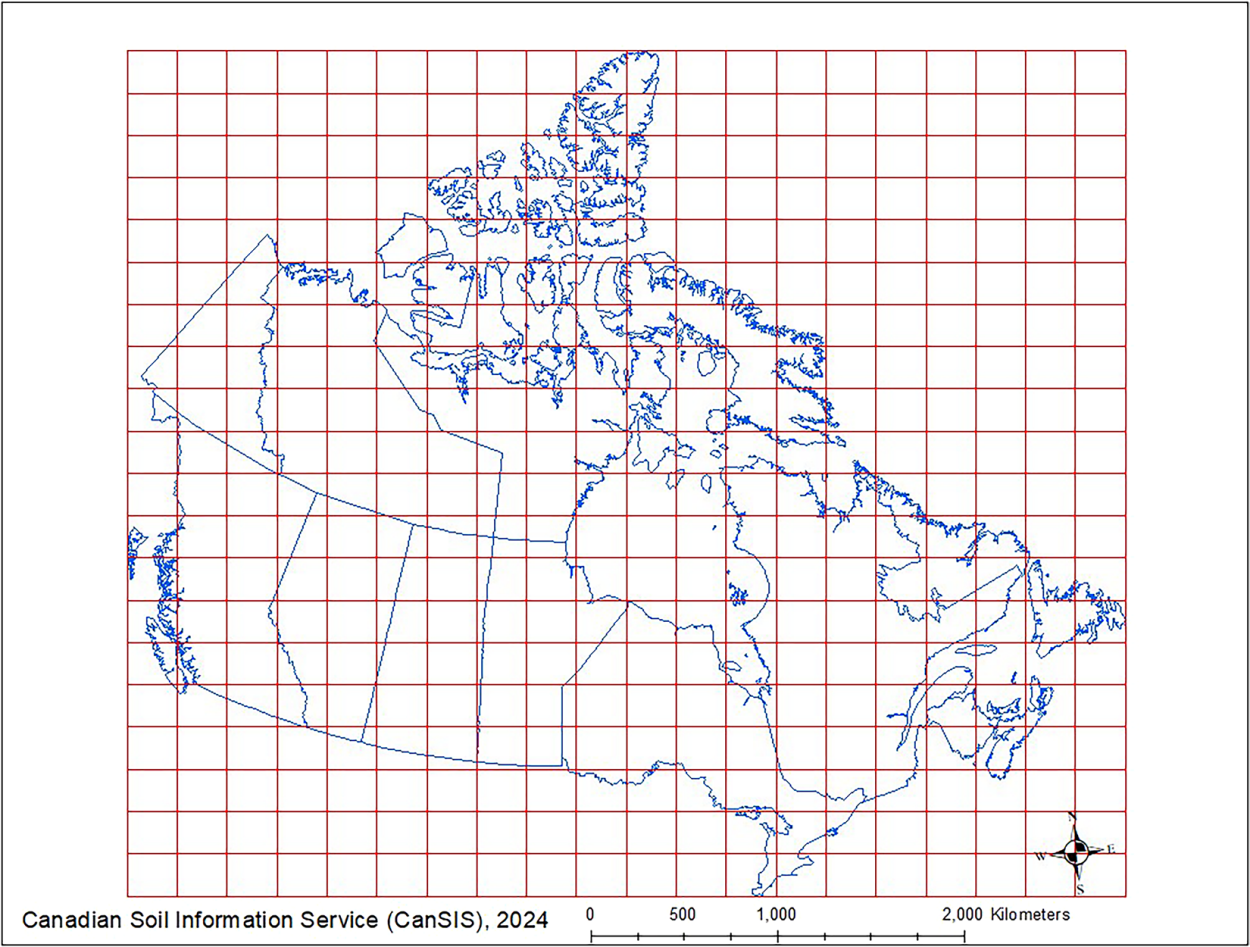
Illustration of tiling system. A systematic tiling system is used both for formulating parallel computing procedures and solving high volume data processing issues.

Tiling and parallel computing solutions were used to make more efficient use of available computing capacity. To cover Canada, 400 tiles were evenly created to subset the underlying data stack used for prediction, bias correction, and uncertainty computations as described in the following sections (Fig. 3). 230 tiles with full or partial overlaps with the landmass of Canada were used in the PSM data pipeline. The Future and Furrr packages for R were used for the implementation of parallel computing^23^.

#### Bias correction and uncertainty measure

Similar to each of the studied machine learners, random forest-based machine learning has limitations. For example, random forest-based machine learning can cause conditional biases which are different from systematic biases^9,11^. Conditional bias in this case means that the variability of predicted attribute values is less than that resulting from training or observation data. That includes the narrowed maximum and minimum range of the observed input data. For example, in addition to spatial variability differences, the range of observed soil bulk density of Canada is between 2.42 g/cm^3^ and 0 g/cm^3^, the predicted data range is between 1.79 g/cm^3^ and 0.27 g/cm^3^. It is an added advancement to include bias correction of random forest machine learner. Based on initial solutions by Zhang and Lu^11^, the bias correction model-1 was used here. The model-1 uses both predictor (independent) variable and response (dependent) variable to correct inference-introduced bias by random forest. For soil class prediction, no bias correction was applied. The uncertainty measure of soil class prediction was calculated from the majority appearance counts in percentage among the prediction iterations.

Under the specifications of the Global Soil Partnership (GSP)^13^, uncertainties of predicted soil class and property data should be attached. However, no specific methods of uncertainty measures are proposed^31^. In this project, for soil properties the 5% and 95% quantiles were generated using QRF algorithms. To present uncertainty intuitively to end users, relative prediction intervals (RPI) were also calculated^31,32^. RPI are the ratio between the prediction interval (PI) and training data confidence interval (TDCI) (Eqs. 2,3,4)^31^. Globally, RPI values below 1 indicate that the predicted values are within the 0.05 and 0.95 quantile range of training data:

2 $$P{I}_{90}={P}_{95}-{P}_{5}$$

3 $${TDC}{I}_{90}={Q}_{95}-{Q}_{5}$$

4 $${RP}{I}_{90}=\frac{P{I}_{90}}{{TDC}{I}_{90}}$$

where

P_95_ is the 95% quantile of the modeled predictions

P_5_ is the 5% quantile of the modeled predictions

Q_95_ is the 95% quantile of the training data

Q_5_ is the 5% quantile of the training data

#### Soil depth data adjustment with depth-to-bedrock data

The outputs of PSM data should be further corrected or adjusted with depth-to-bedrock data. In Canada, there are many locations where soils are shallower than 1 meter. In those places predicted soil attribute values below the depth-to-bedrock are set to “no data”. The input data for this operation include the 100 m soil grids and derived depth-to-bedrock data in meters. The depth-to-bedrock data were compiled using multiple raster and vector data sources (https://sis.agr.gc.ca/cansis/nsdb/psm/depth_to_bedrock_canada_100m.zip). Some abrupt division lines needed to be corrected along with future updates of the national 100 m soil landscape grids because of the multiple data sources and the nature of the compiled depth-to-bedrock data. The outputs represented depth-to-bedrock corrected national 100m soil landscape grids data.

#### Model-based and independent statistical validations

Generally, there are two groups of methods used to validate the outputs of PSM. One group is model-based, which uses spatial stochastic models such as multi-fold cross-validation by randomly splitting training data multiple times during prediction procedures. The other is based on independent probabilistic samples, in which the independent samples can be from specific points or areas. Each group of validation methods can have advantages and disadvantages^15,33^. In this project, indicators of coefficient of determination (R^2^) and root mean squared error (RMSE) from bias-correction QRF models were used. For the whole coverage of Canada, soil organic carbon stocks (T/ha) at 0–30 cm depth derived from this 100 m and the global 250 m grids^5^ were used for correlation analysis.

#### Areal-based statistical validation and data processing

**Fig. 4:**
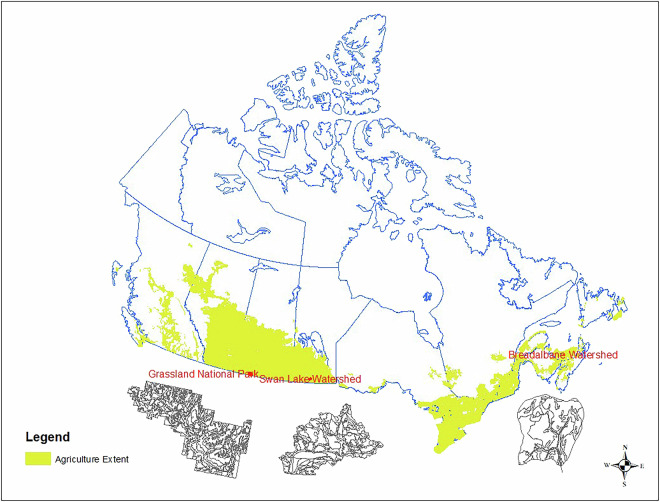
Map of Canada and three study sites. The selected three sites for independent statistical validation all equipped with recent fine resolution predictive soil mapping data.

Canada has seven physiographic regions and a wide variety of soil forming factors such as climate, topography, surficial geological materials, and vegetation covers. Soils across Canada are classified into 10 orders^25^. Although there are national 1:1 million scale soil maps/data, detailed soil surveys were mainly conducted within the agricultural extent of Canada. Less than 7% of land is used for agriculture in Canada (Fig. 4)^3^.

For the predicted soil classes at the Great Group classification level^25^, areal-based statistical validation was conducted with dominant soil types summarized by 1:1 million scale soil landscape of Canada (SLC) version 3.2 polygons (https://sis.agr.gc.ca/cansis/nsdb/slc/v3.2/index.html). Quantity and allocation disagreement statistics were calculated between the SLC and 100 m soil grids based dominant soil types summarized by the SLC polygons^34^.

While the national soil landscape grids were produced with a PSM method for the entirety of Canada in this study, three study sites where detailed soil surveys (https://sis.agr.gc.ca/cansis/nsdb/dss/v3/index.html) and recent predictive soil mapping data exist were selected for further statistical independent validations of this 100 m soil landscape grids data.

In the selected sites, soil properties from soil surveys and various PSM sources were summarized by each of the soil survey polygons^12^. Among the compared soil attributes of different data sources, summary statistics and Pearson correlation coefficients were calculated using R^23^.

#### Swan Lake watershed site

The Swan Lake watershed site is located within a sub-watershed of the Swan Lake basin, Manitoba, Canada. The region receives an annual average of 530 mm of precipitation. The average annual temperature of the region is 1.6 °C and the frost free period ranges from 87 to 110 days between 1991 and 2000 (https://climate.weather.gc.ca/climate_normals/index_e.html#1991, accessed November 25. 2024). The dominant landscapes of the site are flat to rolling topography. Most of this area is under annual crop cover with some under grasslands. The detailed soil surveys of this region are at 1:50,000 scale. From this site, 10 m soil landscape grids data were derived using the same PSM method as the one for this work, with purposively designed point soil samples from 2022.

#### Breadalbane watershed site

The Breadalbane watershed is located in Prince Edward Island (PEI) within the Gulf of St. Lawrence region of Canada. The cool and humid climate is mainly influenced by continental air masses that are humidified and temperature-moderated by the surrounding ocean waters. January and July mean temperatures are −7 °C and 18.7 °C, respectively with an annual mean precipitation of 1100 mm. The frost-free period varies from 100 to 160 days allowing for the cultivation of a wide variety of crops^35,36^. The conventional soil survey data used in this study were based on 1:20,000 scale soil survey. From this site, with purposefully designed point soil samples from 2018, 10 m soil landscape grids data were derived using the same PSM method as the one for the 100 m soil grid development.

#### West Block of the Grasslands National Park site

The West Block of the Grasslands National Park, Saskatchewan, Canada is located within a semi-arid region with an annual mean precipitation of 363 mm between 1991 and 2000. While approximately one third of this total falls as snow, the remainder falls as rain mostly during infrequent heavy summer thunderstorms. From June to August, normal daily mean temperatures range from 15 °C to 18 °C (https://climate.weather.gc.ca/climate_normals/index_e.html#1991, accessed November 25. 2024). In this site, the available conventional soil survey is at a scale of 1:50,000; with purposively designed point soil samples from 2021, 50 m soil landscape grids data were derived using the same PSM method as the one used for this work.

#### Areal data processing and compilation

**Fig. 5:**
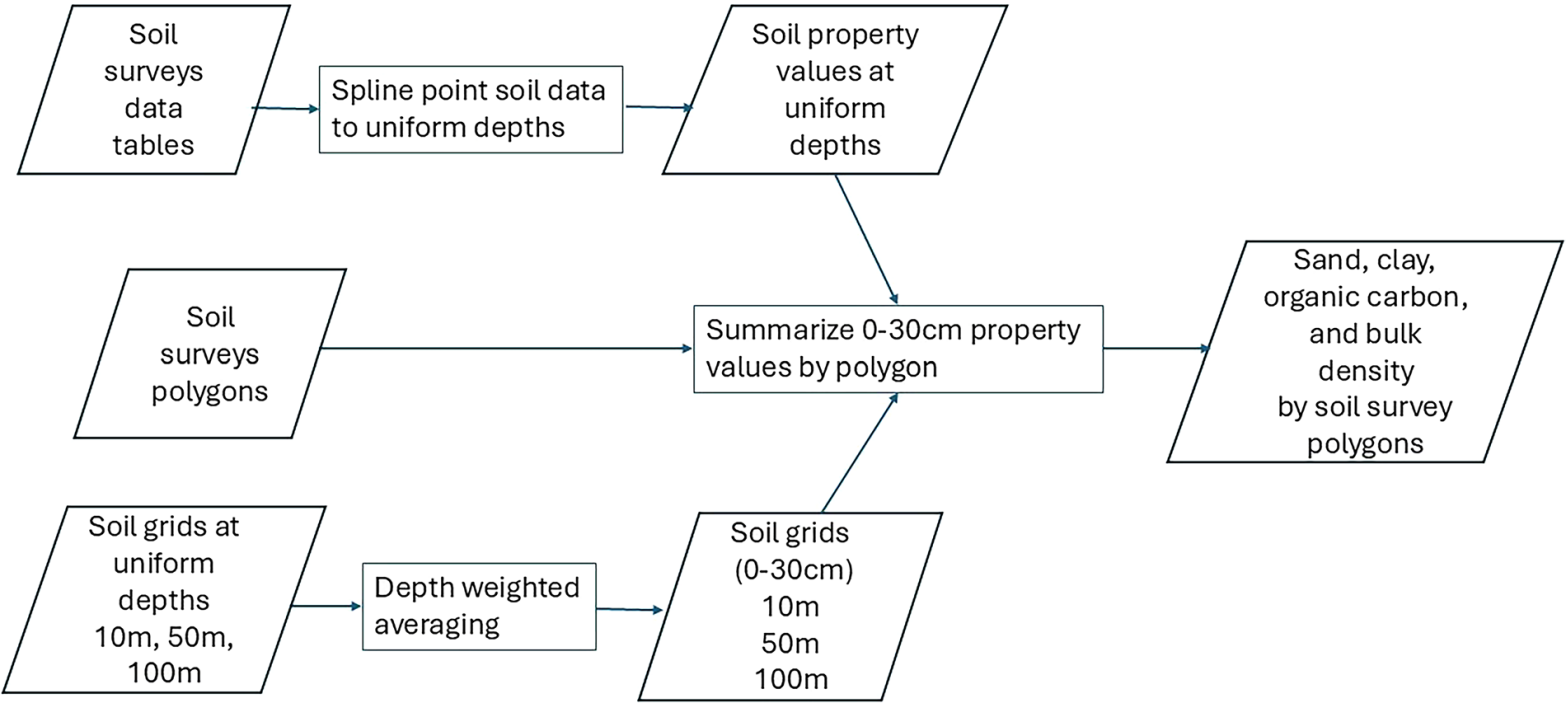
Flow diagram of soil survey polygon-based summary. Different sources of site-specific data were summarized by soil survey polygons before statistical analysis.

The available soil surveys and PSM data come with different scales and resolutions. However, the data structures or data models are common. For the soil surveys, mapped soil areal polygons are associated with polygon attribute table (PAT), soil component table (SCT), soil name table (SNT) and soil layer table (SLT). Within a soil polygon, there can be more than one soil component. Each of the percentile soil components is linked to a unique soil name/type. A soil name/type is linked to multiple records of soil layers. Further details of the soil survey entity model are described in the Canadian Soil Information Service (CanSIS) web site (https://sis.agr.gc.ca/cansis/nsdb/dss/v2/data_model.html). Among the reported soil attributes, soil bulk density (BD) (g/cm^3^), sand (%), clay (%), and soil organic carbon (SOC) (%) were selected for independent evaluation use. Sand and clay data are inherent soil properties. SOC content and BD represent both inherent and human activity influenced soil properties. For example, SOC content at 0–30 cm depends strongly on climate, soil, topography, land use, and management. These soil properties vary greatly across fields and landscapes due these factors. Among the three selected sites, valid soil survey polygons were used for areal data summary and comparison. Figure 5 shows the key steps of soil survey polygon-based data compilation and processing. The final areal data of soil BD, sand content clay content, and SOC content from 0–30 cm depth were used for further summary statistics and correlation coefficient calculations.

All the processed soil survey and soil grids data and codes are provided via a GitHub repository (https://github.com/CanSISPSM/PSM100mCanada).

## Data Records

The 100 m soil landscape grids of Canada data are available at Zenodo^37^. The data set is also accessible via a government public facing web site (link: https://sis.agr.gc.ca/cansis/nsdb/psm/index.html) The primary distribution list includes GeoTiff files for SOC, BD, sand, clay, silt, pH (CaCl_2_ buffered), cation exchange capacity (CEC) and associated quantile uncertainty data at five depth intervals. Further details of the data are provided via data stack metadata (link: https://agriculture.canada.ca/atlas/data_donnees/griddedSoilsCanada/supportdocument_documentdesupport/en/ISO_19131_Soil_Landscape_Grids_of_Canada_100m_%e2%80%93_Data_Product_Specifications.pdf).

## Technical Validation

Table 2 shows coefficients of determination (R^2^) and root mean squared error (RMSE) between the out-of-bag (OOB) predicted and observed data. Overall, the intrinsic statistical validation with R^2^ values range between 0.70 and 0.82 indicates strong performance of the RF with bias correction measures.

**Table 2:** Out of bag accuracy of 100m predictive soil mapping.

Soil properties	R^2^	RMSE
SOC at 0–5 cm depth (%)	0.82	3.44
SOC at 5–15 cm depth (%)	0.82	3.36
SOC at 15–30 cm depth (%)	0.82	3.40
BD at 0–5 cm depth (g/cm^3^)	0.73	0.13
BD at 5–15 cm depth (g/cm^3^)	0.72	0.13
BD at 15–30 cm depth (g/cm^3^)	0.70	0.14
Sand at 0–5 cm depth (%)	0.75	10.31
Sand at 5–15 cm depth (%)	0.76	10.03
Sand at 15–30 cm depth (%)	0.76	10.81
Clay at 0–5 cm depth (%)	0.72	6.41
Clay at 5–15 cm depth (%)	0.71	6.74
Clay at 15–30 cm depth (%)	0.73	7.36

With the 0–30 cm SOC stock(T/ha) data of the 250 m grids^5^ and this 100 m soil grids data, the overall coefficient of determination (R^2^) is 0.49. For the overall accuracy assessment of predicted soil classes, Table 3 shows 62% overall agreement between the predicted and soil survey reported values.

**Table 3:** Statistical accuracy of predicted soil classes.

Accuracy measure	Value %
Overall agreement	62
Overall disagreement	38
Overall quantity disagreement	19
Overall allocation disagreement	19

Among the three independent statistical validation sites, soil survey polygons were used to summarize SOC, BD, sand, and clay contents from available soil surveys with scales ranging from 1:20,000 to 1:50,000, and from soil grids with resolutions ranging from 10 m to 100 m. SOC, BD, sand and clay values at 0–30 cm depth are summarized for the Swan Lake, Breadalbane, and Grassland National Park sites (Tables 4–6). Although the mean values of SOC, BD, sand, and clay by soil survey polygons are generally within expected ranges, there are wider mean value ranges for some attributes among the sites. For example, at the Swan Lake site, the soil survey reported mean sand content was 35.29% which is much lower than 52.84% and 48.67% of the 10 m and 100 m soil grids reported respectively.

**Table 4:** Summary statistics of selected soil attributes, Swan Lake site.

Soil properties at 0–30 cm depth	Mean	Maximum	Minimum	SD
SOC of detailed soil survey (%)	2.11	4.06	0.39	0.97
SOC of 10 m soil grids (%)	2.71	4.22	1.93	0.62
SOC of 100 m soil grids (%)	2.63	3.5	1.83	0.32
BD of detailed soil survey (g/cm^3^)	1.26	1.49	0.32	0.26
BD of 10 m soil grids (g/cm^3^)	1.09	1.19	0.97	0.06
BD of 100 m soil grids (g/cm^3^)	1.27	1.32	1.24	0.02
Sand of detailed soil survey (%)	35.29	55.73	11.88	13.57
Sand of 10 m soil grids (%)	52.84	76.57	37.37	9.71
Sand of 100 m soil grids (%)	48.67	67.07	37.57	6.91
Clay of detailed soil survey (%)	24.58	34.9	17.69	5.2
Clay of 10 m soil grids (%)	20.01	22.14	18.36	1.08
Clay of 100 m soil grids (%)	21.36	26.26	12.79	2.42

**Table 5:** Summary statistics of selected soil attributes, Breadalbane site.

Soil properties at 0–30 cm depth	Mean	Maximum	Minimum	SD
SOC of detailed soil survey (%)	1.7	2.13	0.38	0.52
SOC of 10 m soil grids (%)	2.33	3.22	1.75	0.31
SOC of 100 m soil grids (%)	1.65	2.83	1.32	0.38
BD of detailed soil survey (g/cm^3^)	1.23	1.69	0.71	0.18
BD of 10 m soil grids (g/cm^3^)	0.99	1.21	0.47	0.15
BD of 100 m soil grids (g/cm^3^)	1.21	1.23	1.19	0.01
Sand of detailed soil survey (%)	62.7	83.45	36.02	7.93
Sand of 10 m soil grids (%)	53.92	60.14	48.73	2.74
Sand of 100 m soil grids (%)	58.39	62.75	55.36	1.73
Clay of detailed soil survey (%)	8.61	10.67	2.49	2.35
Clay of 10 m soil grids (%)	9.8	11.96	6.4	1.34
Clay of 100 m soil grids (%)	11.56	12.52	10.17	0.55

**Table 6:** Summary statistics of selected soil attributes, Grassland National Park site.

Soil properties at 0–30 cm depth	Mean	Maximum	Minimum	SD
SOC of detailed soil survey (%)	1.39	1.51	0.99	0.1
SOC of 50 m soil grids (%)	1.9	2.51	1.26	0.19
SOC of 100 m soil grids (%)	2.6	3.35	1.7	0.33
BD of detailed soil survey (g/cm^3^)	1.37	1.4	1.16	0.05
BD of 50 m soil grids (g/cm^3^)	1.09	1.14	0.98	0.02
BD of 100 m soil grids (g/cm^3^)	1.24	1.27	1.19	0.02
Sand of detailed soil survey (%)	33.47	51.33	15.67	5.07
Sand of 50 m soil grids (%)	30.97	36.63	15.15	3.67
Sand of 100 m soil grids (%)	51.57	57.63	42.61	3.3
Clay of detailed soil survey (%)	29.46	43.62	20.72	2.86
Clay of 50 m soil grids (%)	31.72	55.34	24.48	4.07
Clay of 100 m soil grids (%)	16.48	25.11	12.33	3.52

**Fig. 6:**
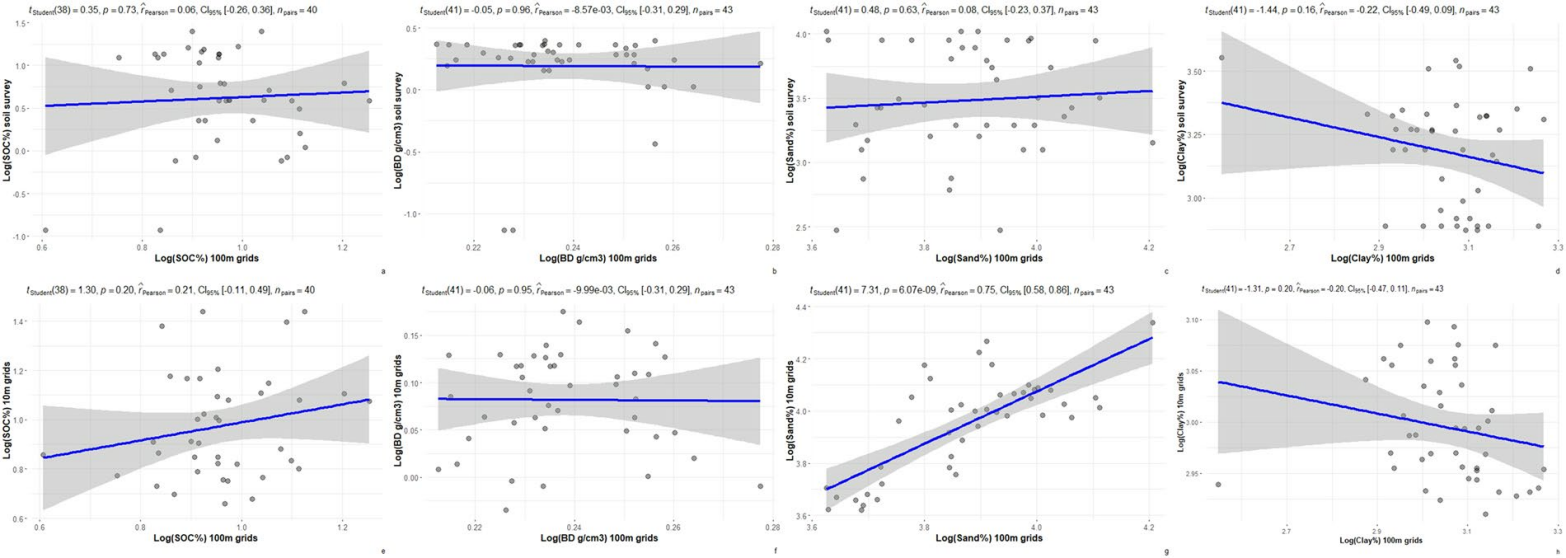
Scatter plots of selected soil properties by soil survey polygons, Swan Lake site.

**Fig. 7:**
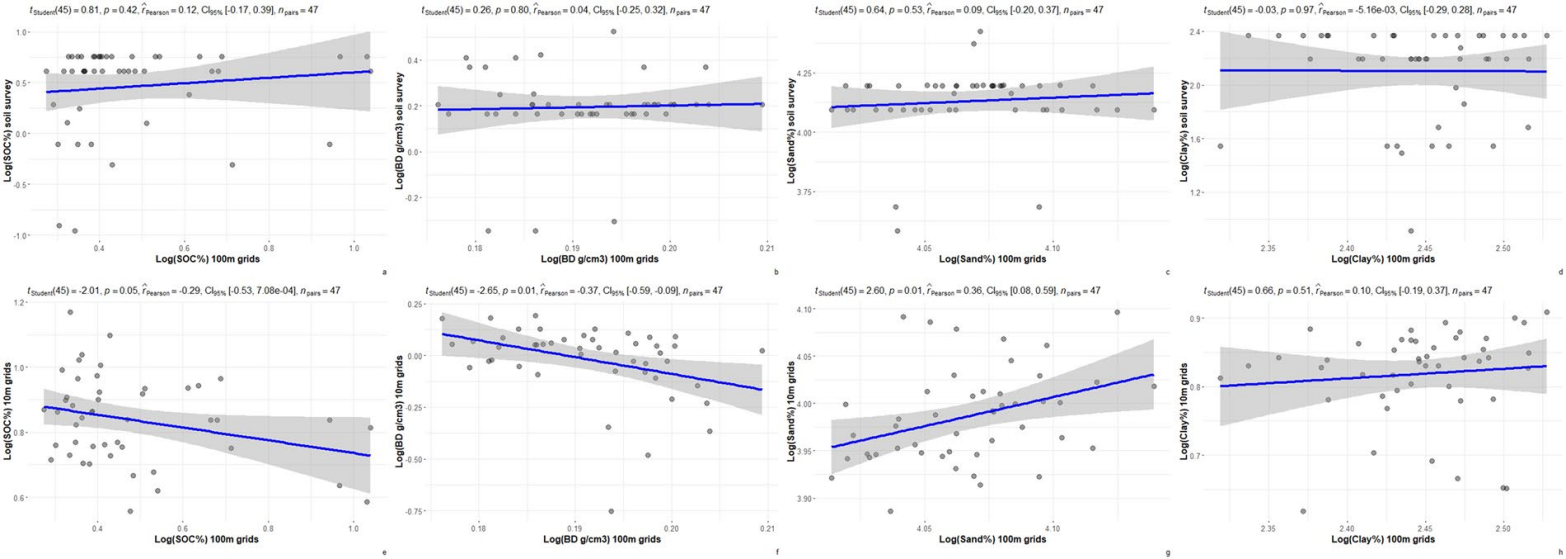
Scatter plots of selected soil properties by soil survey polygons, Breadalbane site.

**Fig. 8:**
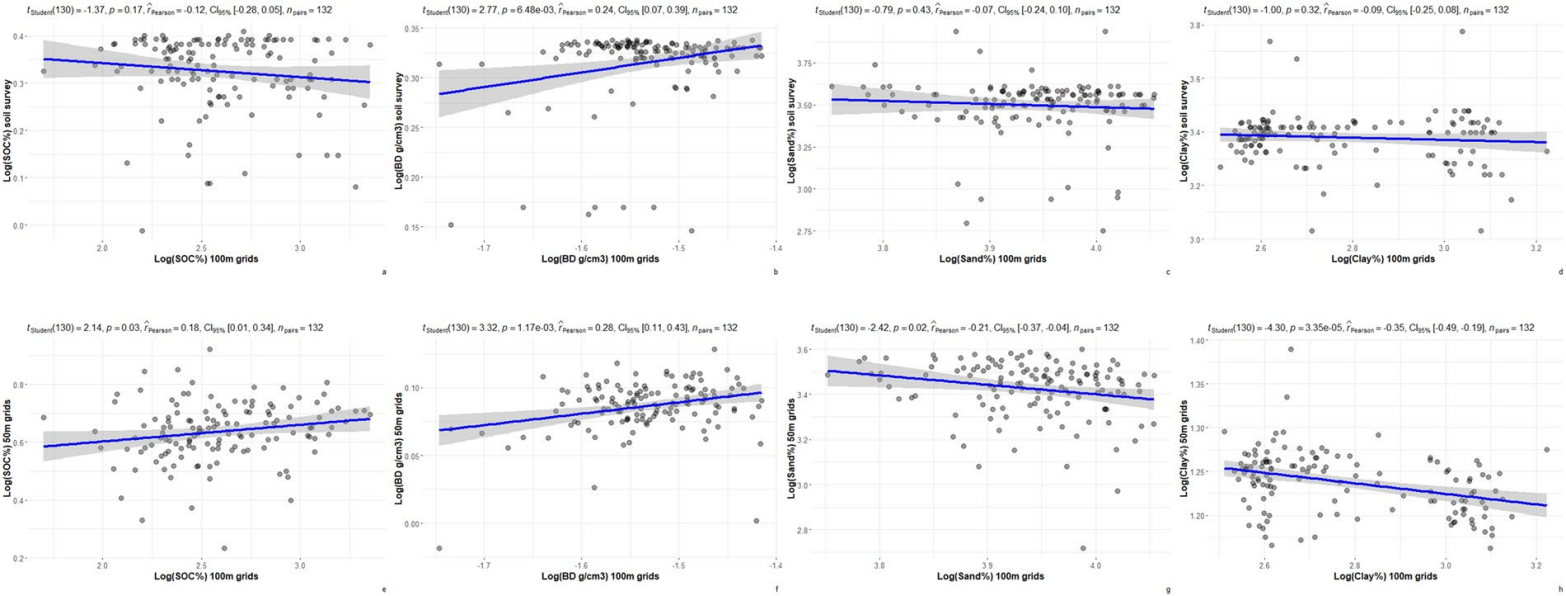
Scatter plots of selected soil properties by soil survey polygons, Grassland National Park site.

Further correlation analysis outcomes are presented in Figs. 6–8. The correlation analysis results presented in Figs. 6–8 show degrees of agreement. For example, in Fig. 8, the soil bulk density of soil surveys are positively correlated to the 50 m and 100 m soil grids. There are also disagreements in the results. For example, in Fig. 8 clay content summarized by soil survey polygons were shown to be negatively correlated between the 50 m and 100 m soil grids.

Legacy soil surveys data were collected 20–30 years ago and are highly generalized. In contrast, the 10 m to 50 m soil grids of the selected sites were more spatially-explicit and based on recent soil samples. Neither the legacy soil survey nor the finer soil grids can offer true validation data for the 100 m soil grids in this case. However, the degrees of disagreement on compared soil properties between the 100 m soil grids and finer resolution data sources indicate that the 100 m soil grids should not be used for field scale applications. For instance, for the Swan Lake site (Fig. 6), predicted SOC and sand contents of 100 m soil grids are positively correlated with the values from finer 10 m soil grids. In contrast, the predicted BD and clay values of 100 m soil landscape grids are negatively correlated with the values from the 10 m soil grids. For Bread Albane (Fig. 7), predicted sand and clay values of 100 m soil grids are positively correlated with the ones of the 10 m soil grids while predicted SOC and BD values are negatively correlated with the ones of 10 m soil grids. For Grassland National Park (Fig. 8), predicted SOC and BD values of the 100 m soil grids are positively correlated with the ones of 50 m soil grids while predicted sand and clay contents of the 100 m soil grids are negatively correlated with the ones of 50 m soil grids. This 100 m soil landscape grid data set is the current version of the ongoing incremental soil grid data development by the Canadian Soil Information Service. With more added ground point data, the accuracy of the 100 m soil grids is expected to improve.

## Usage Notes

Except for the metadata of this published data stack, all the raster data files are in GeoTiff format. Both commercial-off-the-shelf and open-source geographic information system software can be used to read, manipulate, and integrate this data set of files. Given the resolution (100 m) and incremental developmental nature of this data set, the data are suitable for national and regional scale applications and decision-making uses. For applications at watershed and field scales, finer resolution soil grids should be developed and used.

## Data Availability

Along with subset of inputs data, the R code used to create the 100 m soil grids of Canada is available on GitHub (https://github.com/CanSISPSM/PSM100mCanada).
